# Supplementary material for: The interplay between the polar growth determinant DivIVA, the segregation protein ParA, and their novel interaction partner PapM controls the Mycobacterium smegmatis cell cycle by modulation of DivIVA subcellular distribution
Source: Microbiol Spectr. 2023 Nov 15;11(6):e01752-23. doi: 10.1128/spectrum.01752-23 (PMC10714820; doi:10.1128/spectrum.01752-23)
Supplement: Supplemental figures — Fig. S1 to Fig. S7. [file spectrum.01752-23-s0001.pdf]

Supplementary Figures

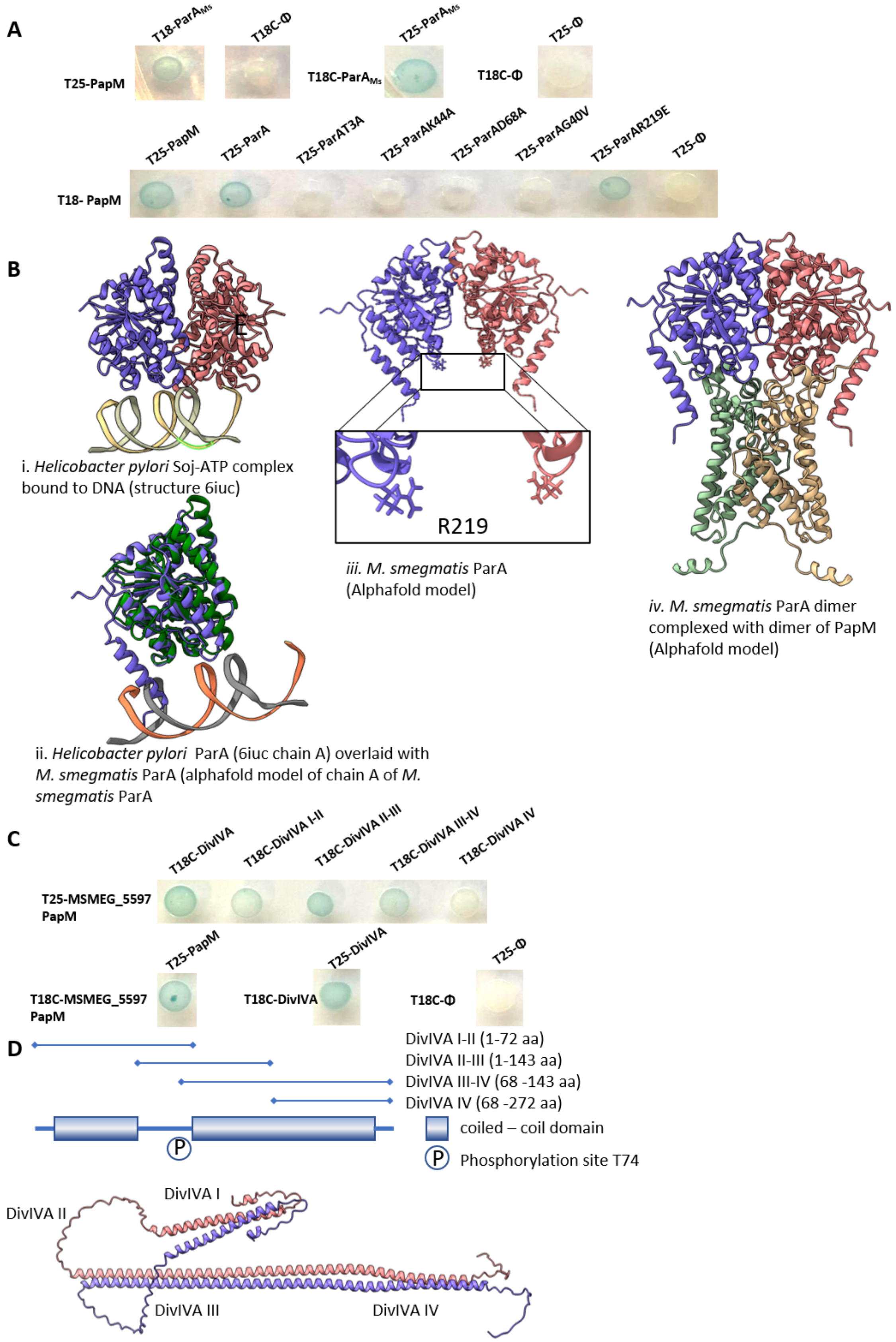

Fig. S1. Analysis of PapM interaction with ParA variants and DivIVA subdomains. a. BTH analysis of ParA-PapM interactions. **b.** Homology modelling and structure prediction of ParA and PapM: i.

Structure of Helicobacter pylori Soj-ATP complex bound to DNA 6iuc (Chu et al. 2019). ii. Chain A of 6iuc overlaid with alphafold model of chain A of ParA of M. smegmatis. The structures overlay with an RMSD of 0.915 Å over 210 pruned atom pairs indicating a conserved core fold. iii Alphafold model of ParA of M. smegmatis as a dimer. The for DNA binding critical residue R219 is magnified. iv Alphafold model of dimer of ParA predicted with dimer of PapM. **c**. BTH analysis of DivIVA-PapM interactions. The blue color of the colony indicates interaction. **d**. The scheme of DivIVA primary structure indicates the domains used for analyses and prediction of DivIVA structure.

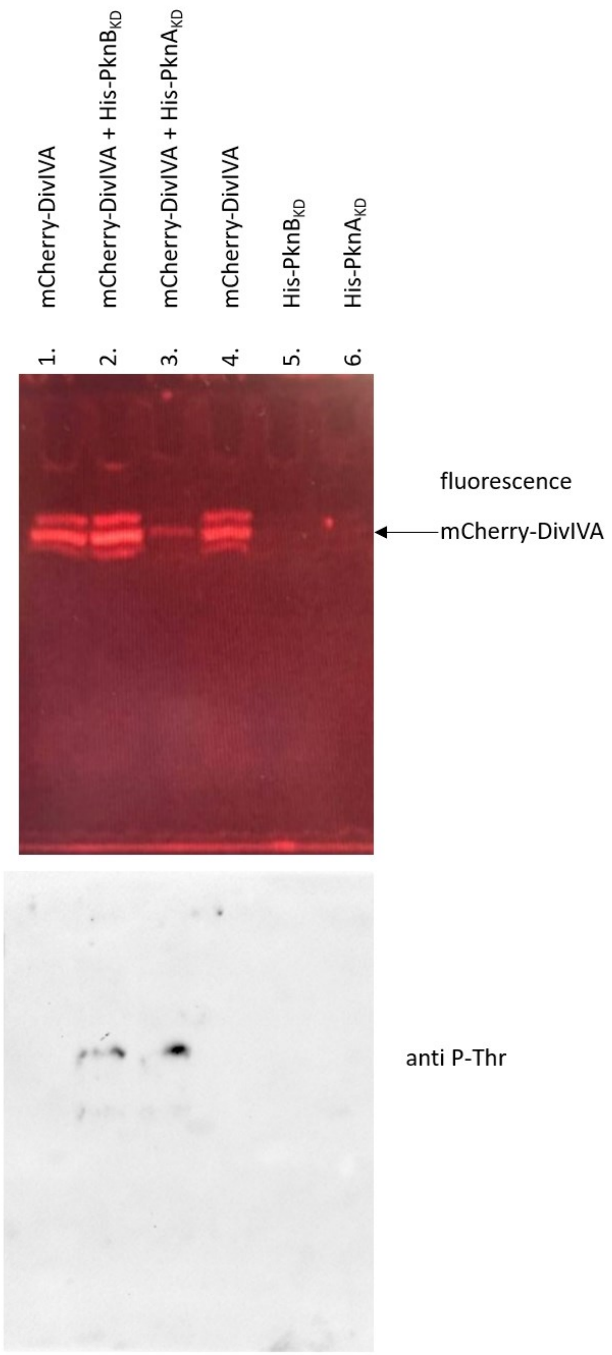

Fig. S2. DivIVA is phosphorylated by PknB and PknA kinase domain in *E coli*. Top panel – mCherry-DivIVA fluorescence in semi-denaturing SDS-PAGE gel (samples were not boiled prior to loading). Multiple bands may suggest protein processing, but since they are present also in the control samples, they were not taken into account. Bottom panel – Western blot with anti-P-Thr antibody confirming mCherry-DivIVA phosphorylation in presence of kinase domain of PknB and PknA. **Lanes:** 1- lysate of the *E. coli* BL21 producing mCherry-DivIVA, 2- lysate of the *E. coli* BL21 producing mCherry-DivIVA and His-PknB$_{KD}$, 3- lysate of the *E. coli* BL21 producing mCherry-DivIVA and His-PknA$_{KD}$, 4- lysate of the *E. coli* BL21 producing mCherry-DivIVA (the same as lane 1), 5- lysate of the *E. coli* BL21 producing His-PknB$_{KD}$, 6- lysate of the *E. coli* BL21 producing His-PknA$_{KD}$.

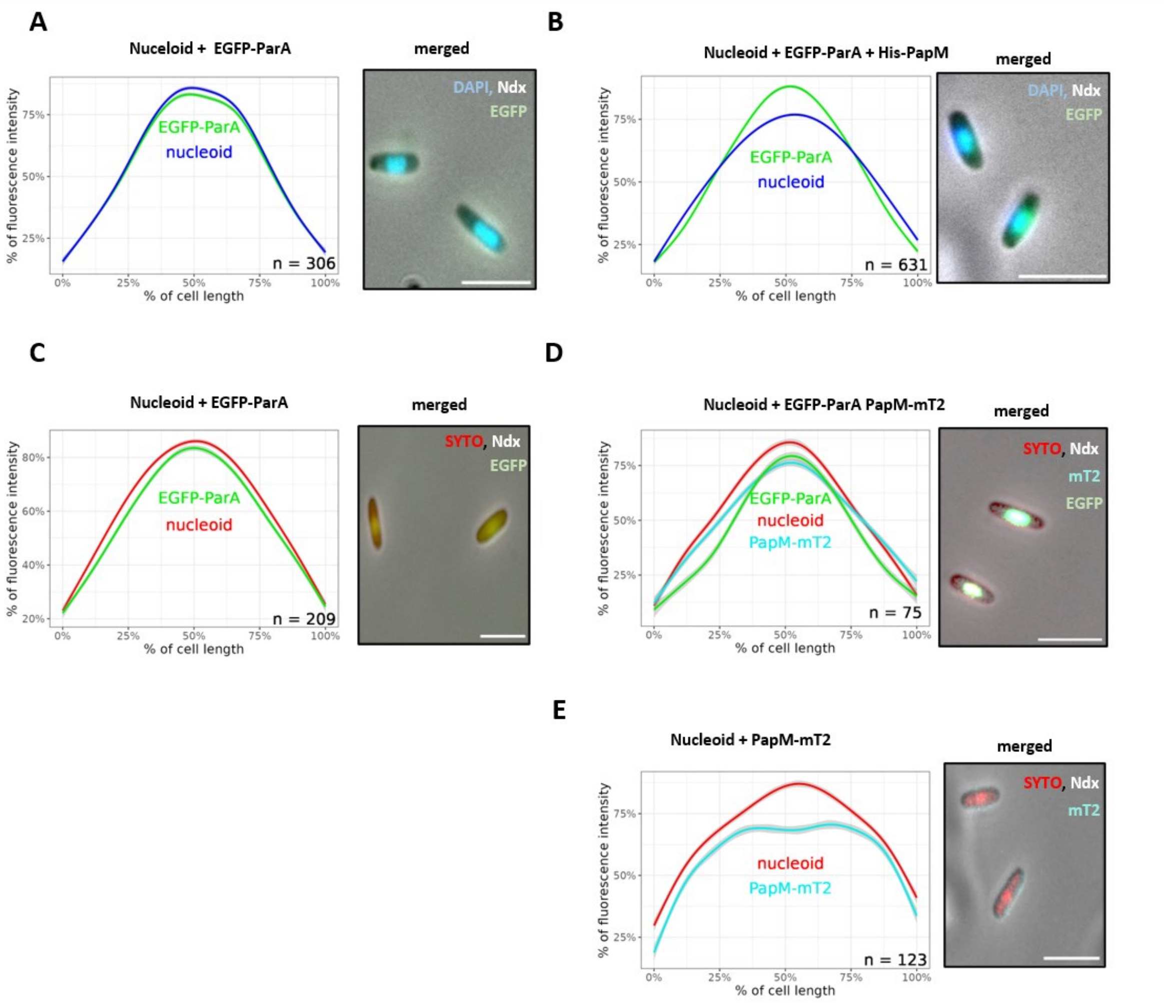

Fig. S3. PapM-mT2 or His-PapM influence on EGFP-ParA colocalization with nucleoid in *E. coli*. A. Colocalization of EGFP-ParA (green) and nucleoid (stained with DAPI – blue). **B**. Colocalization of EGFP-ParA (green) and nucleoid (stained with DAPI – blue) in presence of His-PapM **C**. Colocalization of EGFP-ParA (green) and nucleoid (stained with Syto82 – red) **D**. Colocalization of EGFP-ParA (green) and nucleoid (stained with Syto82 – red) in presence of PapM-mT2 (blue) **E**. Colocalization PapM-mT2 (blue) and nucleoid (stained with Syto82 – red). The cells were treated with nalidixic acid (Ndx) to increase the nucleoid compaction. The left panels show the profiles of fluorescence intensity along the *E. coli* BL21 cells and the right panels show the representative images of *E. coli* cells producing EGFP-ParA (green), PapM-mT2 (blue) and nucleoid stained with SYTO82 (red) or DAPI (blue) (merged fluorescence). The number of the cells used for analysis (n) is indicated in the plots, scale bar 2 µm.

A

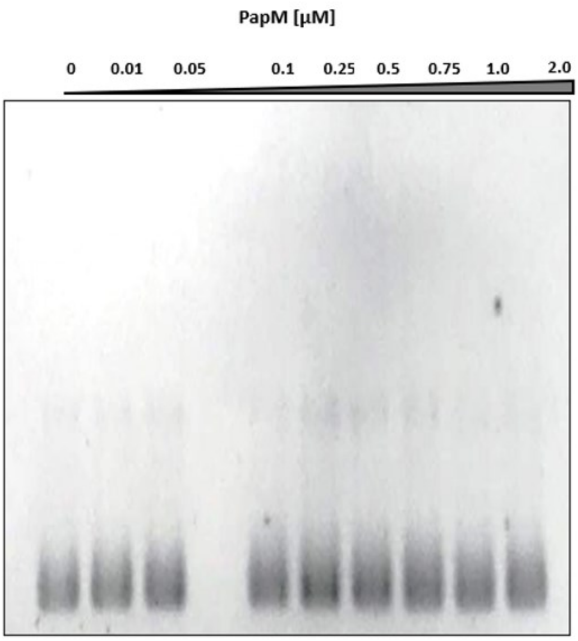

B

```
PapM       2   LDAARELFAERGYDATTVREIAKRAGVSKGALYRYFGSKEELL  44
               LDAA  +F  +GY+  +  ++ +  GV + ALY  FG+KE L
HTH cons. 13   LDAALCVFWRKGYEGASYSDLTQATGVERPALYSAFGNKEALF  55
```

C

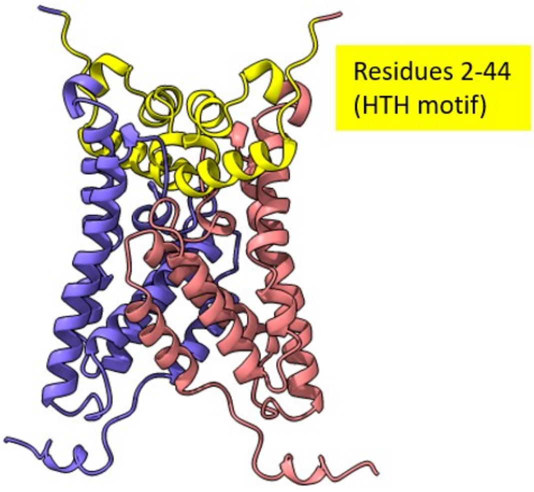

PapM dimer (Alphafold model)

Fig. S4. Analysis of PapM interaction with DNA. A. The EMSA experiment showing lack of PapM binding to pUC19 supercoiled plasmid **B.** The sequence analysis of PapM HTH motif: The consensus sequence of pfam00440: TetR_N aligned to the sequence of PapM. **C.** The alphafold structure of PapM dimer. HTH motif residues are highlighted in yellow.

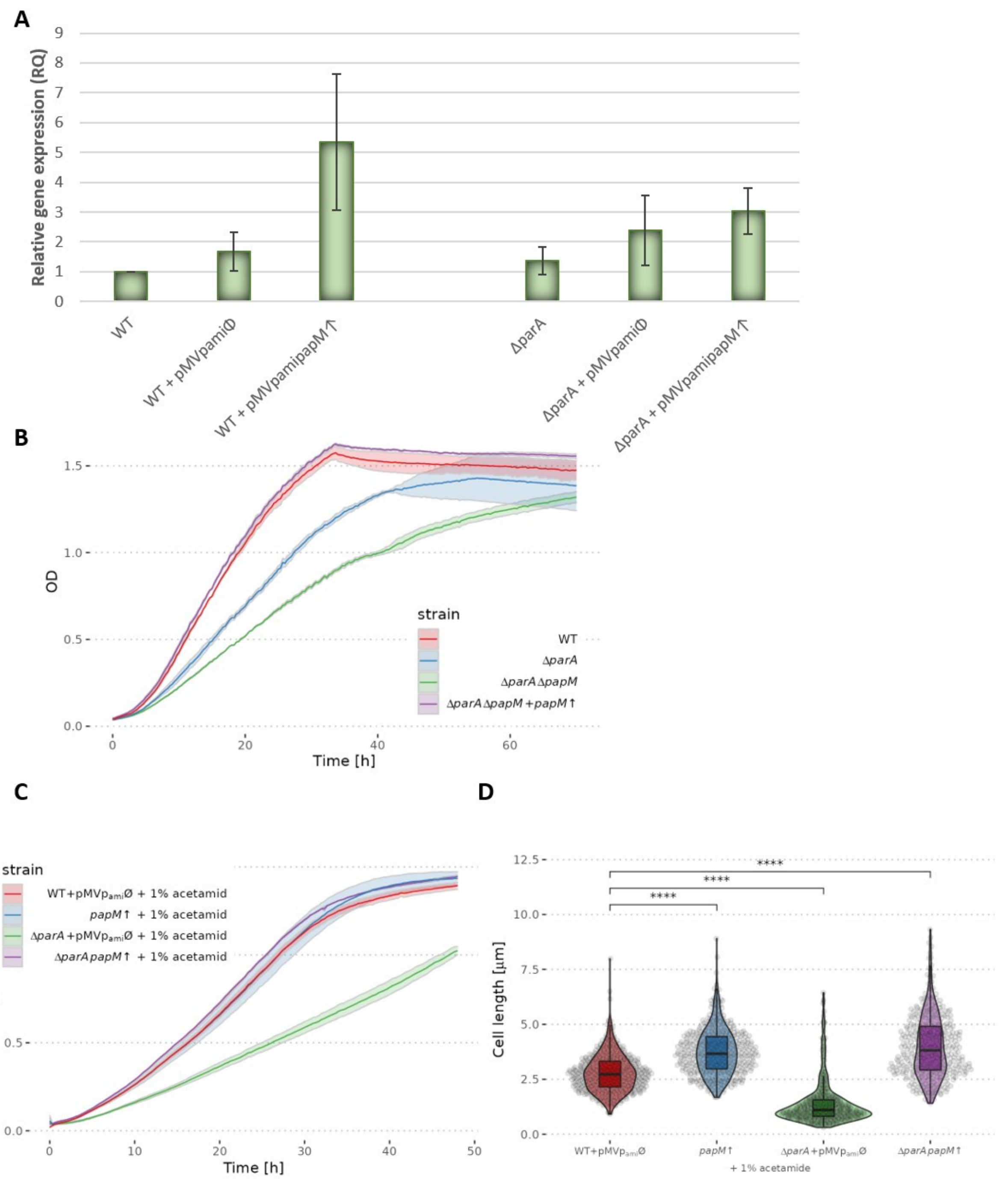

Fig. S5. The impact of *papM* overexpression A. *papM* expression levels in strains containing pMVp$_{\mathrm{ami}}$*papM* in wild type (*papM*↑) and *parA* deletion (Δ*parA papM*↑) background as compared to their parent strains: wild type and Δ*parA*. The data come from 4 independent biological replicates. **B.** Complementation of Δ*papM*, Δ*parA* strain with pMVp$_{\mathrm{ami}}$*papM.* **C.** Growth and **D.** Cell length of the strain overexpressing *papM* in the wild type (*papM*↑) and Δ*parA* (Δ*parA papM*↑) background, 420 and 362 cells measured, respectively) as compared to controls with empty pMVp$_{\mathrm{ami}}$ vector in the wild type (WT+pMVp$_{\mathrm{ami}}$Ø , 487 cells) or Δ*parA* background (Δ*parA*+ pMVp$_{\mathrm{ami}}$Ø, 386 cells) with 1% acetamide induction. The statistical significance between strains determined by Student's t-test (two-sided) with Holm method used for multiple comparisons is marked with asterisks: p-value ≤0.05 (*), ≤0.01 (**) and ≤0.001 (***).

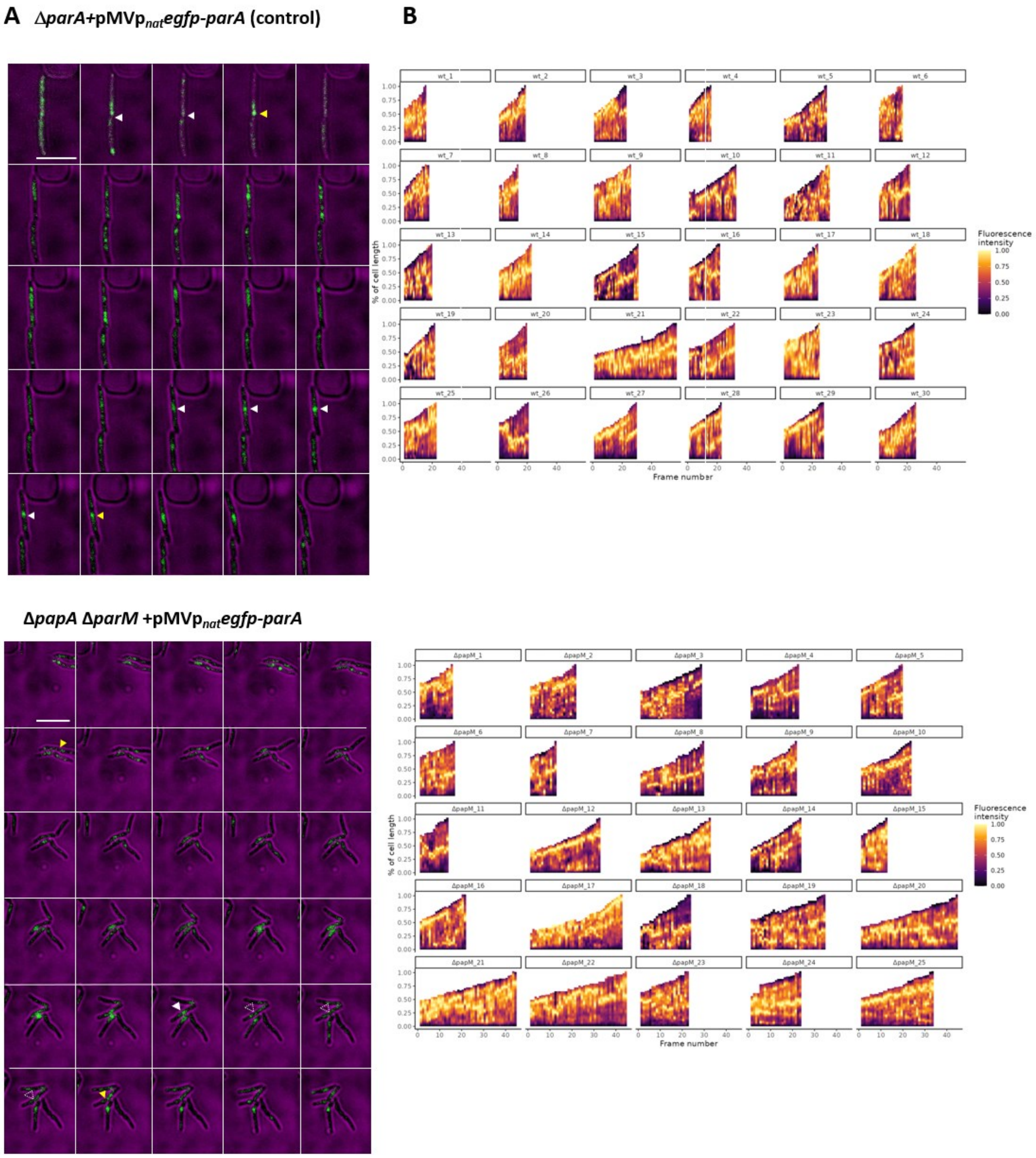

Fig. S6. EGFP-ParA fluorescence profile in the time-lapse analysis. A. The time-lapse images (taken at 10 min intervals) of the *M. smegmatis* cells producing EGFP-ParA in the control strain (Δ*parA*+pMVp$_{nat}$*egfp-parA*) and the Δ*papM* background (Δ*parA*Δ*papM*+pMV306p$_{nat}$*egfp-parA*). The images show EGFP-ParA fluorescence (green) merged with a brightfield image (magenta). The cell division is marked with a yellow arrowhead, the appearance of EGFP-ParA at the site of cell division with white arrowheads (solid or dotted depending on clarity of the signal). Scale bar 5 μm. **B.** The kymographs showing the changes in fluorescence profile in time along each of the analyzed cells. The y-axis illustrates the cell length while time is plotted on the x-axis.

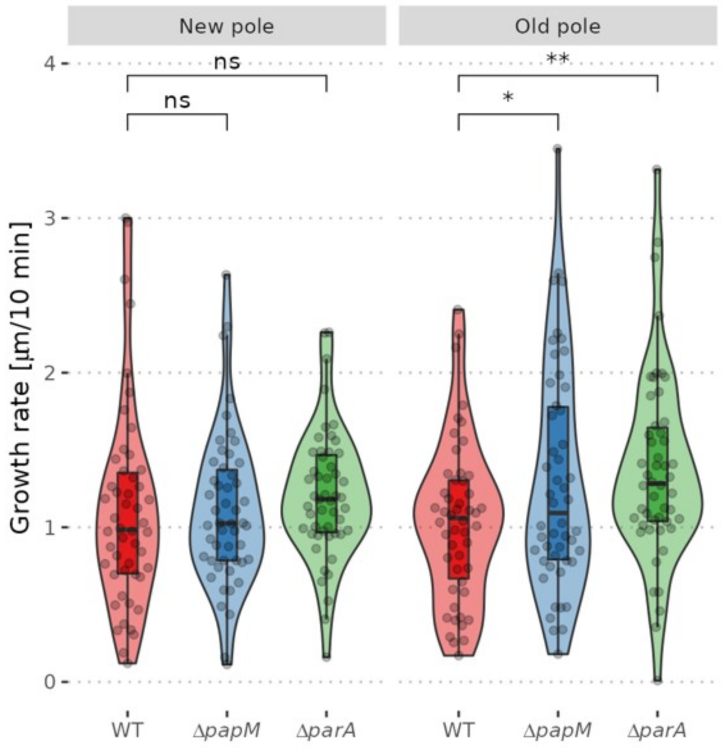

Fig S7. The cell cycle parameters of the wild type, Δ*papM* and Δ*parA* strains A The growth rate of the or new (as indicated) pole inheriting cell in the determined in the time lapse experiment with NADA staining of the wild type, Δ*papM* and Δ*parA* strain (100 cells of each strain analyzed). The data come from two independent biological replicates. The statistical significance between strains determined by Student's t-test (two-sided) with Holm method used for multiple comparisons is marked with asterisks: p-value ≤0.05 (*) and ≤0.01 (**).
