# Supplementary material for: The interplay between the polar growth determinant DivIVA, the segregation protein ParA, and their novel interaction partner PapM controls the Mycobacterium smegmatis cell cycle by modulation of DivIVA subcellular distribution
Source: Microbiol Spectr. 2023 Nov 15;11(6):e01752-23. doi: 10.1128/spectrum.01752-23 (PMC10714820; doi:10.1128/spectrum.01752-23)
Supplement: Supplemental movie legends — Legends for Movies S1 and S2. [file spectrum.01752-23-s0003.pdf]

Matrusiak I. et al. - movie legends

Movie 1. papM deletion alters EGFP-ParA fluorescence dynamics - the time-lapse analysis. The series of time-lapse images (taken at 10 min intervals) showing representative *M. smegmatis* cells producing **(A)** EGFP-ParA in the control strain (Δ*parA*+pMVp$_{nat}$*egfp-parA*) and **(B)** the Δ*papM* background (Δ*parA*Δ*papM*+pMV306p$_{nat}$*egfp-parA*). The images show EGFP-ParA fluorescence (green) merged with a brightfield image (magenta). The cell division is marked with a yellow arrowhead, the appearance of EGFP-ParA at the site of cell division with white arrowheads. Scale bar 5 μm.

Movie 2. The mCherry-DivIVA redistribution during the cell cycle is affected by *parA* and *papM* deletion. The series of time-lapse images (taken at 10 min intervals) showing representative *M. smegmatis* cells mCherry-DivIVA in the **(A)** wild type (WT) control strain, **(B)** Δ*parA* and **(C)** Δ*papM* strains producing mCherry-DivIVA (apart of the wild type DivIVA). The time of DivIVA appearance in the mid-cell is marked with red triangle and the time of new poles separation by black triangle.
