# Supplementary material for: The interplay between the polar growth determinant DivIVA, the segregation protein ParA, and their novel interaction partner PapM controls the Mycobacterium smegmatis cell cycle by modulation of DivIVA subcellular distribution
Source: Microbiol Spectr. 2023 Nov 15;11(6):e01752-23. doi: 10.1128/spectrum.01752-23 (PMC10714820; doi:10.1128/spectrum.01752-23)
Supplement: Table S1 — Constructs used in the study. [file spectrum.01752-23-s0004.pdf]

Matusiak I . et al. supplemental material

Table S1. Constructs used in the study

Constructs	Relevant genotype	Source
Constructs used in *E. coli* BTH system		
pUT18C	pUC replicon, p_{lac} promoter, ampicillin resistance, IPTG-inducible, T18 fragment of *cyaA* gene (encoding 225 to 399 amino acids)	(Karimova et al., 1998)
pKT25	P15A replicon, p_{lac} promoter, kanamycin resistance, IPTG-inducible T25 fragment of *cyaA* gene (encoding 1- 224 amino acids)	(Karimova et al., 1998)
pKT25*parA*	pKT25 derivative containing *M. smegmatis parA* gene	(Ginda et al., 2013)
pKT25*parA*T3A	pKT25 derivative containing *M. smegmatis parA*T3A gene	(Pióro et al., 2019)
pKT25*parA*K44A	pKT25 derivative containing *M. smegmatis parA*K44A gene	(Pióro et al., 2019)
pKT25*parA*D68A	pKT25 derivative containing *M. smegmatis parA*D68A gene	(Pióro et al., 2019)
pKT25*parA*G40V	pKT25 derivative containing *M. smegmatis parA*G40V gene	(Pióro et al., 2019)
pKT25*parA*R219E	pKT25 derivative containing *M. smegmatis parA*R219E gene	(Pióro et al., 2019)
pKT25*parB*	pKT25 derivative containing *M. smegmatis parB* gene	(Ginda et al., 2013)
pUT18C*divIVA*	pUT18C derivative containing *M. smegmatis divIVA* gene	(Ginda et al., 2013)
pUT18C*divIVA I-II*	pUT18C derivative containing a fragment of *M. smegmatis divIVA* gene used for the production of T18C-DivIVAI-II (amino acids 1-72)	(Ginda et al., 2013)
pUT18CdiIVA II-III	pUT18C derivative containing a fragment of *M. smegmatis divIVA* gene used for the production of T18C-DivIVAII - III (amino acids 1-143)	(Ginda et al., 2013)
pUT18C*divIVA III-IV*	pUT18C derivative containing a fragment of *M. smegmatis divIVA* gene used for the production of T18C-DivIVAIII - IV (amino acids 68-272)	(Ginda et al., 2013)
pUT18C*divIVA IV*	pUT18C derivative containing a fragment of *M. smegmatis divIVA* gene used for the production of T18C-DivIVAIV (amino acids 139-272)	(Ginda et al., 2013)
pKT25*divIVA*	pKT25 derivative containing *M. smegmatis divIVA* gene	(Ginda et al., 2013)
pUT18C*msmeg_5597*	pUT18C derivative containing *M. smegmatis msmeg_5597* (*papM*) gene	This study
pKT25*msmeg_5597*	pKT25 derivative containing *M. smegmatis* (*msmeg*_5597) *papM* gene	This study
Constructs used for protein purification		
pGEX-6P-2	pGEX-6P-2 ampicillin resistance, GST-tag vector	Lab stock, University of Wroclaw

pGEX-6P-2*parA*	pGEX-6P-2 derivative containing *M. smegmatis parA* gene	(Jakimowicz et al., 2007)
pET28a	pET28a kanamycin resistance, His-tag vector	Lab stock, University of Wroclaw
pET28a*papM*	pET28a derivative containing *M. smegmatis papM* gene	This study
Constructs used for colocalization microscopy in *E. coli*		
pETDuet-1	ampicillin resistance vector, two multiple cloning sites, the pBR322-derived ColE1 replicon, *lacI* gene	Lab stock, University of Wroclaw
pJP108*divIVA*$_{Ms}$	pJP108 containing *icsA507-620-mcherry* fused with *M. smegmatis divIVA* gene	(Pióro et al., 2019)
pETDuet-*pknB*$_{KD}$	pETDuet-1 derivative containing fragment of *M. smegmatis pknB* gene encoding kinase domain (11-274 aa)	This study
pETDuet-*mcherry-divIVA*	pETDuet-1 derivative containing *icsA507-620-mcherry* fused with *M. smegmatis divIVA* gene	This study
pETDuet-*pknB$_{KD}$ mcherry-divIVA*	pETDuet-1 derivative containing fragment of *M. smegmatis pknB* gene encoding kinase domain (11-274 aa) (MCS1) and *divIVA* gene fused with *mcherry* (MCS2)	This study
pETDuet-*pknB$_{KD}$ mcherry-divIVAT74A*	pETDuet-1 derivative containing fragment of *M. smegmatis pknB* gene encoding kinase domain (11-274 aa) (MCS1) and *divIVAT74A* gene fused with *mcherry* (MCS2)	This study
pETDuet-*ics-mcherry*	pETDuet-1 derivative containing *icsA507-620-mcherry*	This study
pACYC-Duet-1	chloramphenicol resistance vector, two multiple cloning sites, the P15A replicon, *lacI* gene	Lab stock, University of Wroclaw
pACYC-*egfp-parA*	pACYCDuet-1 derivative containing *egfp* gene fused with *M. smegmatis parA* gene	(Pióro et al., 2019)
pACYC-*papM-mT2*	pACYCDuet-1 derivative containing *M. smegmatis papM* gene fused with *mturquoise2*	This study
pACYC-*papM-mT2; egfp-parA*	pACYCDuet-1 derivative containing *M. smegmatis papM* gene fused with *mturquoise2* (MCS1) and *egfp* gene fused with *M. smegmatis parA* gene (MCS2)	This study
Constructs used for *M. smegmatis* modifications		
p2Nil	kanamycin resistance, *oriE*, suicide vector for allelic replacement	(Parish and Stoker, 2000)
pGOAL	ampicillin resistance, *oriE*, selective PacI cassette with *lacZ*, *sacB* and *kanR* genes	(Parish and Stoker, 2000)
p2Nil*ΔpapM*GOAL	p2Nil derivative for deletion of *papM* gene	This study
p2Nil*lsr2-mT2*GOAL	p2Nil containing *mTurquoise2* gene fused with *lsr2*	(Kołodziej et al., 2021)
pMV306p$_{ami}$	pMV306 containing acetamide inducible promoter p$_{ami}$	(Ginda et al., 2013)
pMV306p$_{ami}$*papM*	pMV306p$_{ami}$ derivative containing *M. smegmatis papM* gene	This study

pMVp$_{nat}$*egfp-parA*	pMV306p$_{ami}$ derivative containing *egfp* gene fused with *parA* gene	(Ginda et al., 2013)
pMVp$_{nat}$PA*mcherry-parA*	pMV306p$_{ami}$ containing PA*mcherry* gene fused with *parA* under its native promoter	(Pióro et al., 2019)
pKW08p$_{tet}$	pKW08 containing tetracycline inducible promoter p$_{tet}$	Lab stock, University of Wroclaw
pKW08p$_{tet}$*mcherry-divIVA*	pKW08p$_{tet}$ derivative containing *mcherry* gene fused with *divIVA*	This study

Ginda, K., Bezulska, M., Ziółkiewicz, M., Dziadek, J., Zakrzewska-Czerwińska, J., and Jakimowicz, D. (2013). ParA of Mycobacterium smegmatis co-ordinates chromosome segregation with the cell cycle and interacts with the polar growth determinant DivIVA. *Mol. Microbiol.* 87, 998–1012. doi:10.1111/mmi.12146.

Jakimowicz, D., Brzostek, A., Rumijowska-Galewicz, A., Zydek, P., Dołzbłasz, A., Smulczyk-Krawczyszyn, A., et al. (2007). Characterization of the mycobacterial chromosome segregation protein ParB and identification of its target in Mycobacterium smegmatis. *Microbiology* 153, 4050–4060. doi:10.1099/mic.0.2007/011619-0.

Karimova, G., Pidoux, J., Ullmann, A., and Ladant, D. (1998). A bacterial two-hybrid system based on a reconstituted signal transduction pathway. *Proc. Natl. Acad. Sci.* 95, 5752–5756. doi:10.1073/pnas.95.10.5752.

Kołodziej, M., Trojanowski, D., Bury, K., Hołówka, J., Matysik, W., Kąkolewska, H., et al. (2021). Lsr2, a nucleoid-associated protein influencing mycobacterial cell cycle. *Sci. Rep.* 11, 1–12. doi:10.1038/s41598-021-82295-0.

Parish, T., and Stoker, N. G. (2000). Use of a flexible cassette method to generate a double unmarked Mycobacterium tuberculosis tlyA plcABC mutant by gene replacement. *Microbiology* 146 (Pt 8, 1969–75.

Pióro, M., Małecki, T., Portas, M., Magierowska, I., Trojanowski, D., Sherratt, D., et al. (2019). Competition between DivIVA and the nucleoid for ParA binding promotes segrosome separation and modulates mycobacterial cell elongation. *Mol. Microbiol.* 111, 204–220. doi:10.1111/mmi.14149.
