# Supplementary material for: The interplay between the polar growth determinant DivIVA, the segregation protein ParA, and their novel interaction partner PapM controls the Mycobacterium smegmatis cell cycle by modulation of DivIVA subcellular distribution
Source: Microbiol Spectr. 2023 Nov 15;11(6):e01752-23. doi: 10.1128/spectrum.01752-23 (PMC10714820; doi:10.1128/spectrum.01752-23)
Supplement: Table S2 — Strains used in the study. [file spectrum.01752-23-s0005.pdf]

Matusiak I . et al. supplemental material

Table S2. Strains used in the study

E. coli strains		
DH5α	*F-, endA1, glnV44, thi-1, recA1, relA1, gyrA96, deoR,nupGΦ80dlacZΔM15Δ(lacZYA-argF) U169, hsdR17(rKmK+), λ-*	Lab stock, University of Wroclaw
BTH101	F-, *cya-99, araD139, galE15, galK16, rpsL1 (Str* ^{r}*), hsdR2, mcrA1, mcrB1*	Lab stock, University of Wroclaw
BL21(DE3)	*F-, ompT, gal, dcm, lon, hsdSB(rB- mB-), λ(DE3)*	Lab stock, University of Wroclaw
M. smegmatis strains		
WT	*M. smegmatis* mc^{2} 155	lab stock, University of Wroclaw
KG22	*M. smegmatis* mc^{2} 155 *ΔparA*	(Ginda et al., 2013)
MP24	*M. smegmatis* mc^{2} 155, *attB*L5:: pMV306p$_{ami}$*Ø*	(Pióro et al., 2022)
KG56	*M. smegmatis* mc^{2} 155 *ΔparA,* attBL5:: pMV306p$_{nat}$*egfp–parA*	(Ginda et al., 2013)
DJMP01	*M. smegmatis* mc^{2} 155 *ΔparA,* attBL5:: pMV306p$_{nat}$*PAmcherry–parA*	(Pióro et al., 2019)
IM01	*M. smegmatis* mc^{2} 155 *ΔpapM*	This study
IM02	*M. smegmatis* mc^{2} 155 *ΔparA, ΔpapM*	This study
IM11	*M. smegmatis* mc^{2} 155 *ΔparA,* attBL5::pMVp$_{ami}$Φ	This study
IM12	*M. smegmatis* mc^{2} attBL5::pMVp$_{ami}$*papM*	This study
IM13	*M. smegmatis* mc^{2} 155 *ΔparA,* attBL5::pMVp$_{ami}$*papM*	This study
IM14	*M. smegmatis* mc^{2} 155 *ΔparA, ΔpapM,* attBL5::pMVp$_{nat}$*egfp-parA*	This study
IM15	*M. smegmatis* mc^{2} 155 *ΔparA, ΔpapM,* attBL5:: pMV306p$_{nat}$*PAmcherry–parA*	This study
IM16	*M. smegmatis* mc^{2} attBL5::pKW08p$_{tet}$*mcherry-divIVA*	This study
IM17	*M. smegmatis* mc^{2} *ΔpapM,* attBL5::pKW08p$_{tet}$*mcherry-divIVA*	This study
IM18	*M. smegmatis* mc^{2} *ΔparA,* attBL5::pKW08p$_{tet}$*mcherry-divIVA*	This study
IM19	*M. smegmatis* mc^{2} *ΔparA, ΔpapM,* attBL5::pKW08p$_{tet}$*mcherry-divIVA*	This study
IMPW1	*M. smegmatis* mc^{2} *ΔparA, ΔpapM,* attBL5::pMVp$_{ami}$*papM*	This study
