# Supplementary material for: The interplay between the polar growth determinant DivIVA, the segregation protein ParA, and their novel interaction partner PapM controls the Mycobacterium smegmatis cell cycle by modulation of DivIVA subcellular distribution
Source: Microbiol Spectr. 2023 Nov 15;11(6):e01752-23. doi: 10.1128/spectrum.01752-23 (PMC10714820; doi:10.1128/spectrum.01752-23)
Supplement: Table S3 — Oligonucleotides used in this study. [file spectrum.01752-23-s0006.pdf]

Matusiak I . et al. supplemental material

Table S3. Oligonucleotides used in this study

Name	Sequence 5’ to 3’
PknB_Ms_Fw_HindIII	C**AAGCTT**gATGAGGTCGGCGCGCATC
PknB_Ms_Rv_EcoRI	CT**GAATTC**ATACGAACTCGGTGAGATCCTC
Cherry_Fw_Nde	CC**CATATG**GTGAGCAAGGGCGAGGAGG
Div_revSnaBI	CC**TACGTA**TCAGTTGTTGCCGCGGTTGAACTGG
Ics_Fw_NdeI	**CATATG**AGTACTATTCTGGCAGATAATCTCAGCCATC
Cherry_rvSnaBI	CC**TACGTA**TTACTTGTACAGCTCGTCCATGCCGC
5597_Fw_NcoI	AACTTTAATAAGGAGATATA**CCATGG**GTGGGACGTCATCGCGAGTT
5597_linker_Rv	GCCCGCCGCCGAGCCCGCCGAGCCAGGCGCTTGCGCGTGGCCTTCCCC
linker_mT2_Fw	AAGGCCACGCGCAAGCGCCTGGCTCGGCGGGCTCGGCGGCGGGCT
mt2_Rv_EcoRI	CCTGCAGGCGCGCCGAGCTC**GAATTC**TCACTTGTACAGCTCGTCCA
MSMEG_5597_XbaI_BamHI_Fw	CC**TCTAGA**G**GGATCC**ATGGGACGTCATCGCGAGTT
MSMEG_5597_KpnI_EcoRI_Rv	CC**GGTACC**CG**GAATTC**GCGCTTGCGCGTGGCCTT
Msmeg5597_F1_HindIII_Fw	C**AAGCTT**TATGAAATCGGCCGTTGG
Msmeg5597_F1_BamHI_Rv	C**GGATCC**ACGACCGGTAATAAAACTACC
Msmeg5597_F2_BamHI_Fw	C**GGATCC**TGGGAGTTGGGCGGTACG
Msmeg5597_F2_PacI_Rv	C**TTAATTAA**GCCCTGTCTGCCAGCTTG
5597_pami_XbaI_Fw	CTC**TCTAGA**GTGGGACGTCATCGCGAGTTC
5597_pami_KpnI_Rv	CTT**GGTACC**TCAGCGCTTGCGCGTGGC
RT-PCR3_Fw	ATCGCGAGTTCGATGTGGAC
RT-PCR3_Rv	TACCCTTTGCGCCAGAACAC
pKW08F2	GGTGGTGAGTCATAGTTGC
pKW08RV2	GGATTACACATGACCAACTTC
DivIVA_Ms_Ala_Fw	GCGTCGTCACAGGCGGCATCGTCGATCCCGC
DivIVA_Ms_Ala_Rv	GCGGGATCGACGATGCCGCCTGTGACGACGC

*Boldface type indicates the restriction site.
